# Development and validation of a comprehensive risk warning indicator system for depression in Chinese children and adolescents: A cross-sectional observational study

**DOI:** 10.1097/MD.0000000000044045

**Published:** 2025-08-29

**Authors:** Yan Han, Jingrong Xi, Huizhu Jiang, Chunhong Wei

**Affiliations:** a Huai’an No. 3 People’s Hospital, Huai’an City, Jiangsu, China.

**Keywords:** adolescents, children, Delphi method, depression, early detection, indicator development, mental health screening, pediatric psychiatry, psychometric validation, risk assessment

## Abstract

Depression among children and adolescents has reached epidemic proportions globally, necessitating systematic early detection approaches. Existing screening instruments demonstrate variable accuracy across populations and lack comprehensive and culturally appropriate frameworks for Chinese pediatric populations. This study developed and validated a comprehensive risk warning indicator system for depression detection in Chinese children and adolescents. A mixed-methods approach integrated a systematic literature review, expert consensus methodology, and empirical validation. Twenty distinguished experts from 8 tertiary psychiatric hospitals across 6 provinces participated in 2-round Delphi consultations to refine indicator selection. Psychometric validation employed data from 386 children and adolescents diagnosed with depression (ages 11–18) attending outpatient services. Analytic hierarchy process methodology determined hierarchical indicator weights, while comprehensive reliability and content validity assessments evaluated system performance. The expert consultation achieved 100% response rates with authority coefficients of 0.808 and 0.868 across rounds. The coordination coefficients demonstrated significant consensus (W = 0.131 and 0.142, both *P* < .001). The final system comprised 5 primary domains with 38 secondary indicators: language indicators (weight = 0.347), emotional indicators (0.251), behavioral indicators (0.190), stress event indicators (0.144), and history/genetic history (0.068). Psychometric validation revealed robust reliability (overall Kuder–Richardson Formula 20 = 0.811; split-half = 0.745; test–retest ICC = 0.832) and exceptional content validity (Item-Level Content Validity Index: 0.881–1.000; Scale-Level Content Validity Index Average = 0.995; Scale-Level Content Validity Index Universal Agreement = 0.832). This scientifically rigorous, culturally appropriate indicator system demonstrates excellent psychometric properties and practical applicability for early depression detection across clinical, educational, and community settings, potentially improving prevention and intervention outcomes in vulnerable youth populations.

## 1. Introduction

The global landscape of pediatric mental health has undergone profound transformation in recent decades, with mounting evidence revealing an unprecedented crisis in the psychological well-being of children and adolescents worldwide. Contemporary research demonstrates alarming increases in mental health disorders among youth populations, with emergency department consultations for acute psychopathological symptoms exhibiting significant linear trends, particularly following the COVID-19 pandemic.^[1,2]^ This escalating mental health crisis has become one of the most pressing public health challenges of the 21st century, demanding immediate attention from healthcare systems, educational institutions, and policymakers across diverse socioeconomic and cultural contexts.^[3]^

Depression, as the most prevalent and debilitating psychiatric disorder affecting children and adolescents, represents a critical component of this expanding mental health epidemic. Recent systematic reviews and meta-analyses reveal substantial variability in depression prevalence across different populations and assessment methodologies, with pooled estimates for major depressive disorder ranging from 0.71% in childhood to significantly higher rates in adolescent populations.^[4,5]^ Longitudinal population-based studies indicate point prevalence rates approaching 10% among children in grades 6 to 10, with 12-month prevalence rates of approximately 3% and incidence rates of 4.5% over 12-month periods.^[6]^ These figures represent considerable increases from historical baselines, with some regions documenting near-doubling of severe depression rates among socioeconomically disadvantaged adolescents over the past 2 decades.^[7]^

The clinical significance of these epidemiological trends extends far beyond mere statistical observations, as depression in childhood and adolescence is associated with profound immediate and long-term consequences for cognitive development, academic achievement, social functioning, and overall quality of life. Contemporary research emphasizes that depression among youth populations frequently manifests as a complex constellation of symptoms that may differ substantially from adult presentations, often incorporating somatic complaints, behavioral changes, and academic difficulties that can obscure diagnostic clarity.^[8,9]^ Furthermore, the digital age has introduced novel risk factors and manifestation patterns, with emerging evidence suggesting that technology-mediated social interactions and digital media exposure may contribute to the evolving phenotype of adolescent depression.^[3]^

The imperative for early identification and intervention has become increasingly urgent as mounting evidence demonstrates that untreated depression in youth populations frequently persists into adulthood, contributing to increased risks of suicide, substance abuse, academic failure, and long-term psychiatric comorbidity. Global burden of disease analyses reveal that depressive disorders among youth aged 10 to 29 years have demonstrated consistent upward trends from 1990 to 2021, with disability-adjusted life years continuing to increase across diverse geographical regions and socioeconomic strata.^[10]^ Recent data from high-income countries indicate that depressive symptoms among high school students with health-risk behaviors show particularly concerning trajectories, suggesting that vulnerable populations may be experiencing disproportionate mental health deterioration.^[11]^

Despite growing recognition of this crisis, significant gaps persist in the availability of validated, culturally appropriate, and practically applicable tools for early depression detection in pediatric populations. Existing screening instruments often demonstrate variable sensitivity and specificity across different demographic groups, with limited validation in non-Western populations and insufficient consideration of culturally specific manifestation patterns. Meta-analyses examining mental health problems during the COVID-19 pandemic revealed pooled prevalence rates of 29% for depression and 26% for anxiety among children and adolescents, highlighting both the magnitude of the problem and the urgent need for systematic identification approaches.^[12–14]^

The Chinese context presents unique challenges and opportunities for advancing pediatric depression detection capabilities. According to the 2023 China National Mental Health Development Report, mental health literacy rates among children and adolescents remain critically low at 6.4%, representing the lowest rates among all surveyed demographic groups.^[12]^ This limited mental health awareness, combined with cultural factors that may influence symptom expression and help-seeking behaviors, necessitates the development of assessment tools that are specifically designed and validated for Chinese pediatric populations. Furthermore, China’s emphasis on preventive mental health approaches, as outlined in national mental health policies, provides a supportive framework for implementing systematic early detection initiatives.^[2]^

Current research on depression risk assessment in children and adolescents has predominantly focused on isolated risk factors or single-domain approaches, lacking the comprehensive perspective necessary to capture the multifaceted nature of depression etiology and manifestation. International efforts, such as the IDEA project’s work on depression risk prediction across different continental contexts, have demonstrated the feasibility and importance of developing systematic, multi-domain assessment approaches. However, the cultural specificity of depression presentation patterns, combined with unique healthcare delivery systems and educational structures, necessitates locally developed and validated assessment frameworks that can accommodate diverse manifestation patterns while maintaining scientific rigor.^[15–17]^

This investigation addresses these critical gaps by developing a comprehensive, scientifically rigorous risk warning indicator system specifically designed for depression detection in Chinese children and adolescents. The study systematically integrates linguistic, emotional, behavioral, and environmental factors into a unified assessment framework, translating complex psychological phenomena into observable, measurable indicators that can be readily applied across clinical, educational, and community settings. By employing rigorous consensus methodology combined with empirical validation, this research aims to provide healthcare professionals, educators, and families with practical tools for early depression identification, ultimately contributing to improved prevention and intervention outcomes for vulnerable youth populations.

The significance of this work extends beyond immediate clinical applications to encompass broader public health implications for systematic mental health promotion and early intervention strategies. As healthcare systems worldwide grapple with increasing demands for pediatric mental health services, validated screening tools that enable non-specialist identification of at-risk youth represent critical components of comprehensive prevention frameworks. This study’s emphasis on practical applicability, cultural appropriateness, and scientific validity positions it to make substantial contributions to global efforts in addressing the pediatric mental health crisis while providing a foundation for future research and clinical innovation in depression prevention and early intervention.

## 2. Research methods

### 2.1. Study design and ethical considerations

This cross-sectional study employed a mixed-methods approach combining systematic literature review, expert consultation using the Delphi method, and psychometric validation to develop a comprehensive risk warning indicator system for depression in children and adolescents. This study received full ethical approval from the Institutional Ethics Committee of Huai’an No. 3 People’s Hospital (Ethics Approval Number: 2023-206, approved March 15, 2023). All procedures were conducted in strict accordance with Declaration of Helsinki principles and national regulatory requirements for human subjects research. Written informed consent was obtained from all adult participants. For participants under 16 years, parental/guardian consent was secured alongside participant assent. Adolescents aged 16 to 18 years provided informed consent with additional assent documentation. All consent processes included comprehensive explanation of study procedures, potential risks, benefits, and withdrawal rights.

### 2.2. Research team composition and qualifications

The multidisciplinary research team comprised 8 members with complementary expertise in psychology, health management, education, and biostatistics. The team included 1 doctoral-level researcher, 1 master’s degree holder, and 6 bachelor’s degree holders, with academic ranks distributed as 4 senior professionals, 3 associate senior professionals, and 1 mid-level professional. Team members had a mean clinical experience of 12.3 ± 4.7 years in child and adolescent mental health, ensuring adequate expertise for indicator development. The team was strategically divided into a design group responsible for research protocol development, expert selection, and tool validation, and an execution group managing literature review, data collection, and statistical analysis.

### 2.3. Phase 1: systematic literature review and initial indicator development

#### 2.3.1. Search strategy and selection criteria

A comprehensive literature search was conducted across multiple databases including CNKI, Wanfang, VIP, PubMed, ScienceDirect, and SpringerLink, covering publications from January 2016 to December 2024. The search strategy employed both Medical Subject Headings (MeSH) terms and free-text keywords, with search terms combined using Boolean operators (AND, OR). Chinese search terms included “抑郁症” (Depression), “抑郁障碍” (Depressive disorders), “儿童” (Children), “青少年” (Adolescents), “预警指标” (Early warning indicators), and “危险因素” (Risk factors). English search terms encompassed “depression,” “depressive disorders,” “children,” “adolescents,” “early warning indicators,” and “risk factors.”

#### 2.3.2. Inclusion criteria

- Chinese literature published in core journals indexed in CSCD, CSSCI, CSTPCD, or Peking University Core Journals Catalog (2016–2024).
- English literature published in SCI or SSCI-indexed journals (2016–2024).
- Studies with sample sizes ≥ 100 participants.
- Studies reporting quantitative data on risk factors or predictive indicators.

#### 2.3.3. Exclusion criteria

- Full text unavailable after institutional library access and interlibrary loan requests.
- Studies with methodological quality scores below acceptable thresholds as assessed by 2 independent reviewers.
- Duplicate publications, conference abstracts, or direct translations without original content.
- Studies focusing exclusively on adult populations or specific comorbid conditions.

Two independent reviewers conducted the initial screening based on titles and abstracts, with disagreements resolved through discussion with a third reviewer. Full-text review was performed using standardized data extraction forms, and methodological quality was assessed using the Newcastle–Ottawa Scale for observational studies.

### 2.4. Expert panel formation and initial indicator development

Following the systematic review, 4 senior psychologists (associate senior rank or above) specializing in child and adolescent depression from the hospital’s psychiatric department participated in structured brainstorming sessions. [These sessions were conducted using nominal group technique principles, with each expert independently generating indicators before group discussion and consensus building.] The research team synthesized and refined the discussion outcomes, resulting in a preliminary indicator framework comprising 7 primary domains (language, behavior, emotions, physiology, stress events, medical history and genetic history, interpersonal relationships, and social functioning) and 30 secondary indicators.

### 2.5. Phase 2: Delphi expert consultation process^[18]^

#### 2.5.1. Expert selection and characteristics

Expert participants were selected through purposive sampling based on predetermined criteria ensuring both expertise and geographical representation. A minimum of 15 experts was targeted based on sample size recommendations for Delphi studies in healthcare research.

#### 2.5.2. Inclusion criteria

- Current employment in tertiary psychiatric hospitals with specialized child and adolescent services.
- Minimum 10 years of clinical or research experience in child and adolescent psychiatry or psychology.
- Bachelor’s degree or higher educational qualification.
- Senior professional title or equivalent academic standing.
- Voluntary participation with commitment to complete both consultation rounds.

#### 2.5.3. Exclusion criteria

- Self-rated familiarity as “less familiar” or “unfamiliar” with the study domain.
- Withdrawal from the consultation process before completion.
- Inability to provide detailed rationale for indicator ratings.

Twenty experts from 6 provinces and municipalities (Jiangsu, Shandong, Shanghai, Hunan, Guangdong, and Beijing) were recruited from 8 tertiary psychiatric hospitals. The expert panel included 12 clinical medical experts, 6 nursing management experts, and 2 clinical nursing experts, with a gender distribution of 9 males and 11 females. Experts had a mean age of 48.47 ± 5.60 years and average working experience of 27.71 ± 7.24 years, ensuring adequate expertise and experience diversity.

### 2.6. Consultation questionnaire development

The consultation instrument comprised 4 comprehensive sections designed to capture both quantitative ratings and qualitative feedback:

*Section 1:* consultation explanation provided detailed information about study objectives, methodological approach, and completion instructions. This section included definitions of key terms and examples of indicator applications to ensure consistent interpretation across experts.

*Section 2*: expert background information collected demographic and professional characteristics including gender, age, education level, professional title, current position, research specialization, years of experience, departmental affiliation, and contact information.

*Section 3*: expert self-assessment form required experts to evaluate their familiarity with the study content and assess 4 dimensions influencing their judgment capacity. The self-assessment employed validated scales for expertise evaluation, with familiarity rated on a 5-point scale from “very familiar” (1.0) to “unfamiliar” (0.2). Judgment basis dimensions were weighted according to their relative importance: work experience (weights: 0.5, 0.4, 0.3), theoretical analysis (0.3, 0.2, 0.1), literature familiarity (0.1, 0.1, 0.5), and intuitive judgment (0.1, 0.1, 0.05).

*Section 4*: indicator evaluation table presented each proposed indicator for comprehensive assessment. Experts evaluated scientific validity, appropriateness, categorical fit, and importance using a 5-point Likert scale (5 = very important, 4 = important, 3 = moderately important, 2 = slightly important, 1 = not important). Each indicator included operational definitions and clinical examples to enhance rating consistency. Open-ended comment sections accompanied each indicator to capture qualitative feedback and suggestions for refinement.

### 2.7. Consultation implementation and analysis

The research team distributed consultation questionnaires via secure electronic platforms (WeChat and email) with a 1-week completion deadline. Reminder notifications were sent after 3 and 5 days to optimize response rates. Two consultation rounds were conducted, with the first round involving 10 experts (7 medical experts and 3 nursing experts) and the second round engaging a different cohort of 10 experts (5 medical experts and 5 nursing experts).

Between consultation rounds, the research team conducted 2 structured group discussions lasting 2 to 3 hours each, with detailed minutes recorded and analyzed using thematic analysis principles. Indicators were systematically screened using predetermined criteria: mean importance rating > 3.5, coefficient of variation < 0.25, and full-score ratio > 20%. These thresholds were established based on established Delphi methodology guidelines and previous indicator development studies in healthcare settings.

### 2.8. Phase 3: psychometric validation study

#### 2.8.1. Participant selection and characteristics

A convenience sample of 386 patients was recruited from the outpatient clinic of Huai’an No. 3 People’s Hospital between April and June 2024. Participants were children and adolescents aged 11 to 18 years with confirmed depression diagnoses according to Diagnostic and Statistical Manual of Mental Disorders, 5th Edition criteria. Diagnoses were established by board-certified child and adolescent psychiatrists using structured clinical interviews (MINI-KID) and confirmed through multidisciplinary team review.

#### 2.8.2. Inclusion criteria for validation study

- Age 11 to 18 years at time of assessment.
- Primary diagnosis of major depressive disorder, persistent depressive disorder, or other specified depressive disorder according to Diagnostic and Statistical Manual of Mental Disorders, 5th Edition criteria.
- Ability to provide informed consent/assent.
- Adequate verbal communication skills for assessment completion.

#### 2.8.3. Exclusion criteria

- Presence of acute psychotic symptoms or severe cognitive impairment.
- Current substance uses disorder as primary diagnosis.
- Inability to complete assessment due to severe symptom severity.

### 2.9. Data collection procedures

Trained research assistants, blinded to the study hypotheses, conducted all assessments in private clinical settings using standardized protocols. Each assessment session lasted approximately 45 to 60 minutes, with breaks provided as needed. For participants with limited verbal communication abilities, assessments were conducted through structured observation and caregiver report using validated proxy measures.

The indicator assessment employed a binary scoring system with “yes” responses assigned a value of 1 and “no” responses assigned a value of 0. Inter-rater reliability was established through independent dual coding of 10% of assessments (n = 39), achieving Cohen kappa coefficients > 0.80 for all indicators.

### 2.10. Content validity assessment

Seven experts randomly selected from the consultation panel participated in content validity evaluation using a structured assessment form. These experts were different from those involved in the initial Delphi rounds to ensure independent evaluation. Relevance of each indicator was rated on a 4-point scale ranging from 1 (“very irrelevant”) to 4 (“very relevant”). Content validity ratios were calculated using Lawshe method, with critical values adjusted for the number of expert raters.

### 2.11. Statistical analysis

All statistical analyses were performed using SPSS version 26.0 software (IBM Corp., Armonk) with significance levels set at α = 0.05. Missing data patterns were analyzed, and multiple imputation was employed for missing values representing < 5% of total data points. Expert consultation metrics included calculation of enthusiasm coefficients (questionnaire recovery rates), authority coefficients (Ca), combining judgment basis (Cs) and familiarity scores (Cf), and coordination coefficients using Kendall coefficient of concordance.

Reliability analysis included internal consistency assessment using Kuder–Richardson Formula 20 (KR-20) for dichotomous items, with coefficients ≥ 0.70 considered acceptable. Split-half reliability was calculated using Spearman–Brown prophecy formula, with coefficients ≥ 0.60 deemed satisfactory.

Content validity indices were calculated at both item level (Item-Level Content Validity Index [I-CVI]) and scale level, with I-CVI values ≥ 0.78 considered acceptable. Scale-level content validity was assessed using both average (Scale-Level Content Validity Index Average [S-CVI/Ave] ≥ 0.90) and universal agreement (Scale-Level Content Validity Index Universal Agreement [S-CVI/UA] ≥ 0.80) approaches. Bootstrap confidence intervals (95% CI) were calculated for all validity and reliability coefficients to assess precision of estimates.

Indicator weights were determined using the analytic hierarchy process (AHP) implemented in YAAHP version 0.3 software (Jeff Zhang, China), with combined weights calculated using the product method to integrate expert judgments across consultation rounds. Consistency ratios were calculated for all pairwise comparison matrices, with ratios < 0.10 considered acceptable for weight derivation.

## 3. Results

### 3.1. Expert panel characteristics and consultation process

**Figure 1.**
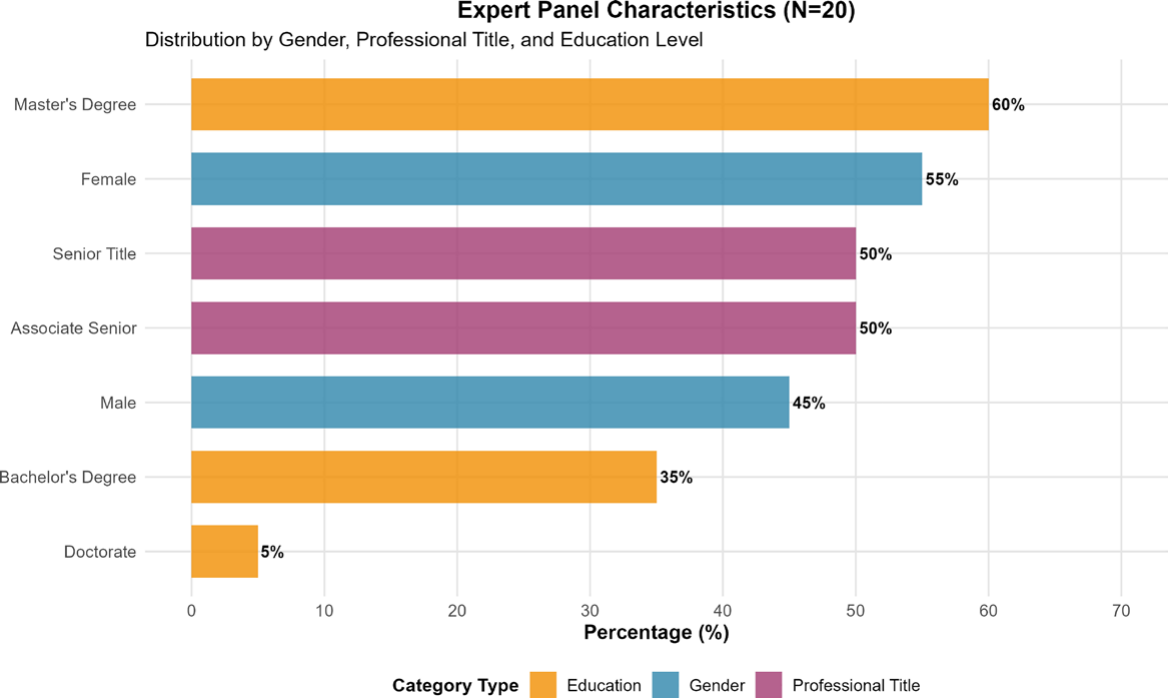
Expert panel characteristics and composition (N = 20). Distribution of expert panel members by gender, professional title, and educational qualifications across 6 provinces and 8 tertiary psychiatric hospitals. The panel demonstrated balanced representation across demographic and professional categories, ensuring comprehensive expertise in child and adolescent mental health.

The expert consultation process successfully engaged 20 distinguished professionals from 6 provinces and municipalities across China, representing 8 tertiary psychiatric hospitals specializing in child and adolescent mental health services. The expert panel demonstrated exceptional diversity in both geographical representation and professional expertise, encompassing clinical medical experts (n = 12, 60%), nursing management experts (n = 6, 30%), and clinical nursing experts (n = 2, 10%). Gender distribution was well-balanced with 9 male (45%) and 11 female (55%) participants. The panel’s extensive experience was reflected in their mean age of 48.47 ± 5.60 years and substantial clinical experience averaging 27.71 ± 7.24 years in child and adolescent psychiatry and psychology (Table 1, Fig. 1).

**Table 1:** Expert panel characteristics and consultation metrics.

Characteristics	Round 1 (n = 10)	Round 2 (n = 10)	Overall (N = 20)
Demographics			
Age, mean ± SD, yr	47.8 ± 6.2	49.1 ± 5.1	48.47 ± 5.60
Gender, n (%)			
Male	4 (40.0)	5 (50.0)	9 (45.0)
Female	6 (60.0)	5 (50.0)	11 (55.0)
Professional characteristics			
Experience, mean ± SD, yr	26.4 ± 8.1	29.0 ± 6.2	27.71 ± 7.24
Professional category, n (%)			
Clinical medical	7 (70.0)	5 (50.0)	12 (60.0)
Nursing management	3 (30.0)	3 (30.0)	6 (30.0)
Clinical nursing	0 (0.0)	2 (20.0)	2 (10.0)
Professional title, n (%)			
Senior	6 (60.0)	4 (40.0)	10 (50.0)
Associate senior	4 (40.0)	6 (60.0)	10 (50.0)
Education level, n (%)			
Doctorate	1 (10.0)	0 (0.0)	1 (5.0)
Master’s	6 (60.0)	6 (60.0)	12 (60.0)
Bachelor’s	3 (30.0)	4 (40.0)	7 (35.0)
Consultation metrics			
Response rate (%)	100.0	100.0	100.0
Experts providing suggestions, n (%)	6 (60.0)	6 (60.0)	12 (60.0)
Authority coefficient (Ca)	0.808	0.868	0.838
Judgment basis (Cs)	0.868	0.899	0.884
Familiarity (Cf)	0.796	0.837	0.817
Coordination coefficient (W)	0.131***	0.142***	–
Chi-square (χ^2^)	371.893	363.077	–

*** *P* < .001.

The educational qualifications of the expert panel were robust, with 60% holding master’s degrees, 35% holding bachelor’s degrees, and 5% possessing doctoral qualifications. Professional rankings demonstrated senior expertise, with 50% holding senior professional titles and 50% holding associate senior titles (Table 1, Fig. 1).

The 2-round consultation process was strategically designed to ensure comprehensive evaluation and refinement of the indicator system. The first round engaged 10 experts comprising 7 medical specialists and 3 nursing professionals, while the second round involved a different cohort of 10 experts with 5 medical specialists and 5 nursing professionals. This alternating expert composition was deliberately implemented to minimize bias and ensure independent evaluation across consultation rounds.

### 3.2. Expert engagement and methodological rigor

The consultation process demonstrated exceptional expert engagement and methodological rigor across both rounds. All distributed questionnaires (n = 10 per round) were returned within the 7-day completion timeframe, achieving a 100% effective response rate that substantially exceeded the established threshold of 70% for adequate expert enthusiasm. The mean completion time for questionnaires was 4.2 ± 1.8 days, indicating thorough consideration rather than hasty responses. Notably, 60% of experts (n = 12) provided detailed qualitative feedback and specific suggestions for indicator refinement, demonstrating their substantial investment in the study’s scientific rigor.

**Figure 2.**
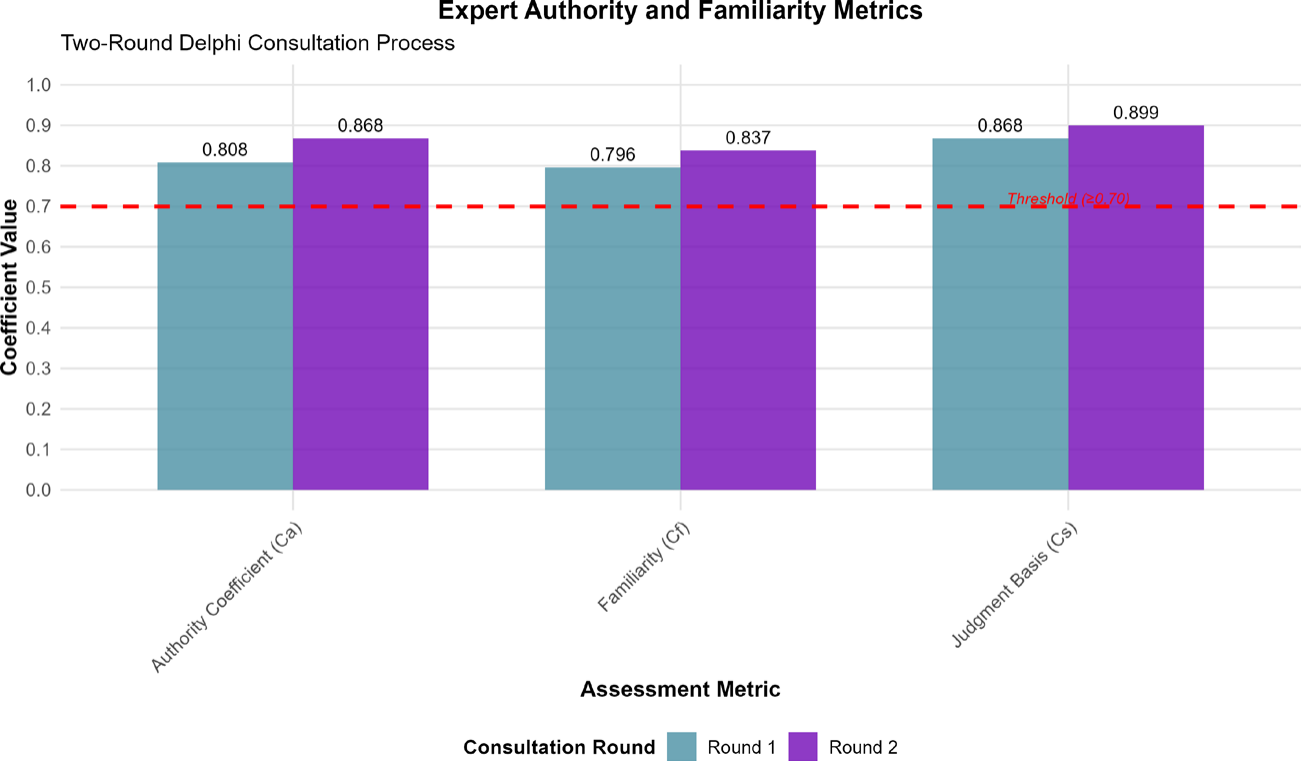
Expert authority and familiarity metrics across consultation rounds. Authority coefficients (Ca), judgment basis (Cs), and familiarity (Cf) scores for both Delphi consultation rounds. All metrics exceeded the established threshold of ≥ 0.70 (red dashed line), with progressive improvement from Round 1 to Round 2, demonstrating high expert competence and subject matter expertise.

The expert Ca revealed consistently high levels of professional competence and subject matter expertise. In the first consultation round, the Ca reached 0.808, with Cs at 0.868, and Cf at 0.796. The second round demonstrated even stronger authority metrics with Ca = 0.868, Cs = 0.899, and Cf = 0.837. These values substantially exceeded the established threshold of Ca ≥ 0.70 for acceptable expert authority, with both rounds achieving authority levels characteristic of high-quality Delphi studies (Fig. 2).

Expert opinion coordination was assessed through coefficient of variation and Kendall coefficient of concordance analyses. All indicators demonstrated coefficient of variation values ≤ 0.25, indicating minimal dispersion and strong consensus among expert opinions. The mean coefficient of variation across all indicators was 0.18 ± 0.04 in round 1 and 0.16 ± 0.03 in round 2, demonstrating progressive convergence of expert opinions. The coordination coefficients (W) were statistically significant for both rounds: W = 0.131 (χ² = 371.893, *P* < .001) in round 1 and W = 0.142 (χ² = 363.077, *P* < .001) in round 2, confirming consistent and credible expert consensus (Table 1).

### 3.3. Indicator development and refinement process

#### 3.3.1. First round consultation outcomes

The initial expert consultation yielded comprehensive feedback that substantially refined the indicator framework. Three experts emphasized the critical importance of focusing on practical, controllable factors within children’s primary environments, specifically home and school settings. While past medical history and genetic predisposition were initially considered for removal due to their uncontrollable nature, expert consensus supported their retention based on their established relevance to depression risk assessment and early warning capabilities.

Quantitative analysis of first-round responses revealed that 23 of the original 30 secondary indicators met the predetermined screening criteria (mean importance rating > 3.5, coefficient of variation < 0.25, and full-score ratio > 20%). Seven indicators were eliminated due to insufficient expert consensus, primarily those related to demographic and socioeconomic factors.

Significant structural modifications emerged from expert recommendations. Two experts proposed consolidating physiological indicators with language indicators, recognizing that children with depression frequently express physical discomfort through verbal communication. This integration was adopted following extensive group discussion and theoretical justification. This consolidation reduced redundancy while maintaining comprehensive coverage of somatic symptom expression patterns.

Specific indicator refinements included strategic splitting of complex constructs to enhance precision. The indicator “devaluation of self-worth and self-blame or guilt” was divided into 2 distinct secondary indicators to capture different psychological processes. Similarly, “frequent complaints of physical discomfort” was merged with “no organic physical disease found through medical examination but frequent complaints of discomfort” to create a more comprehensive somatic symptom indicator.

Novel indicators were introduced based on expert clinical experience, including “what is the least painful way to die” under language indicators, “sleep disturbances and eating disorders” under behavioral indicators, and “body image anxiety” under stress event indicators. These additions reflected contemporary understanding of adolescent depression presentation.

### 3.4. Second round consultation and final indicator system

The second consultation round involved 4 experts who provided targeted feedback leading to final system refinement. The language indicator “frequently expressing inner distress and conveying suicidal tendencies uncontrollably” was strategically separated into 2 distinct secondary indicators to differentiate between general emotional expression and specific suicidal communication. This separation enhanced the clinical utility of the system by providing more precise risk stratification capabilities.

Behavioral indicators were expanded through the addition of “perceptual disturbances” and “inconsistencies between behavior and inner feelings,” reflecting expert recognition of the complex phenomenology of adolescent depression. These additions increased the behavioral domain from 8 to 10 secondary indicators, enhancing the comprehensiveness of behavioral assessment. The emotional indicators domain was strengthened with “physical symptoms often accompanying low mood,” acknowledging the frequent somatic presentation of depression in pediatric populations.

The stress events category was enhanced with “environmental adaptation issues,” recognizing the significant impact of ecological transitions on adolescent mental health. This addition increased the stress events domain to 9 secondary indicators, providing more comprehensive coverage of environmental risk factors.

### 3.5. Psychometric validation results

#### 3.5.1. Reliability assessment

The psychometric validation employed data from 386 children and adolescents diagnosed with depression who received outpatient services between June and September 2024. The sample demographic characteristics included 198 females (51.3%) and 188 males (48.7%), with a mean age of 14.6 ± 2.1 years, representing a well-balanced cohort for validation purposes.

Internal consistency analysis revealed robust reliability characteristics. The overall KR-20 coefficient achieved 0.811, substantially exceeding the established threshold of ≥ 0.70 for acceptable reliability. Individual domain reliability coefficients ranged from 0.724 (history and genetic history) to 0.856 (language indicators), with all domains meeting acceptable reliability standards (Table 2). The split-half reliability coefficient reached 0.745, surpassing the required threshold of ≥ 0.60 and confirming the instrument’s temporal stability.

**Table 2:** Final risk warning indicator system with weights and validation metrics.

Primary domain	Weight	Secondary indicators	Combined weight	I-CVI	Domain KR-20
Language indicators	0.347				0.856
		Uncontrollably revealing suicidal ideation	0.084	1.000	
		Frequent complaints of general fatigue	0.062	0.943	
		Lack of life goals and motivation	0.062	1.000	
		Expressing inner distress frequently	0.045	1.000	
		Inquiring about painless death methods	0.038	0.881	
		Frequent somatic complaints without organic cause	0.032	1.000	
		Self-devaluation expressions	0.024	1.000	
		Self-blame and guilt expressions	0.020	1.000	
		Verbal expressions of hopelessness	0.020	0.943	
Emotional indicators	0.251				0.823
		Morning low, evening high mood pattern	0.105	1.000	
		Persistent emotional downturn	0.068	1.000	
		Emotional lability and irritability	0.042	1.000	
		Anhedonia and loss of interest	0.036	1.000	
		Physical symptoms accompanying low mood	0.028	0.914	
		Feelings of worthlessness	0.025	1.000	
		Concentration difficulties	0.022	0.943	
		Emotional numbness	0.018	1.000	
Behavioral indicators	0.190				0.798
		Self-harming behavior	0.035	1.000	
		Sleep disturbances and eating disorders	0.032	1.000	
		Social withdrawal and isolation	0.028	1.000	
		Academic performance decline	0.025	0.914	
		Perceptual disturbances	0.022	0.886	
		Inconsistencies between behavior and inner feelings	0.020	1.000	
		Decreased personal hygiene	0.018	1.000	
		Agitation or psychomotor retardation	0.015	0.943	
		Substance use behaviors	0.012	1.000	
		Risk-taking behaviors	0.010	0.886	
Stress event indicators	0.144				0.767
		Being criticized or punished	0.031	1.000	
		Experiencing school bullying	0.024	1.000	
		Family conflict or dysfunction	0.022	1.000	
		Environmental adaptation issues	0.019	0.886	
		Academic pressure and failure	0.016	1.000	
		Peer relationship problems	0.014	0.943	
		Body image anxiety	0.012	1.000	
		Loss of significant relationships	0.010	1.000	
		Financial family stress	0.008	0.914	
History and genetic history	0.068				0.724
		Direct relatives with depression	0.034	1.000	
		History of trauma and abuse	0.021	1.000	
		Previous suicide attempts or self-harm	0.013	0.943	

Overall system metrics:

• Total secondary indicators: 38.

• Overall KR-20: 0.811.

• Split-half reliability: 0.745.

• S-CVI/Ave: 0.995.

• S-CVI/UA: 0.832.

S-CVI/Ave = Scale-Level Content Validity Index Average, S-CVI/UA = Scale-Level Content Validity Index Universal Agreement.

**Figure 3.**
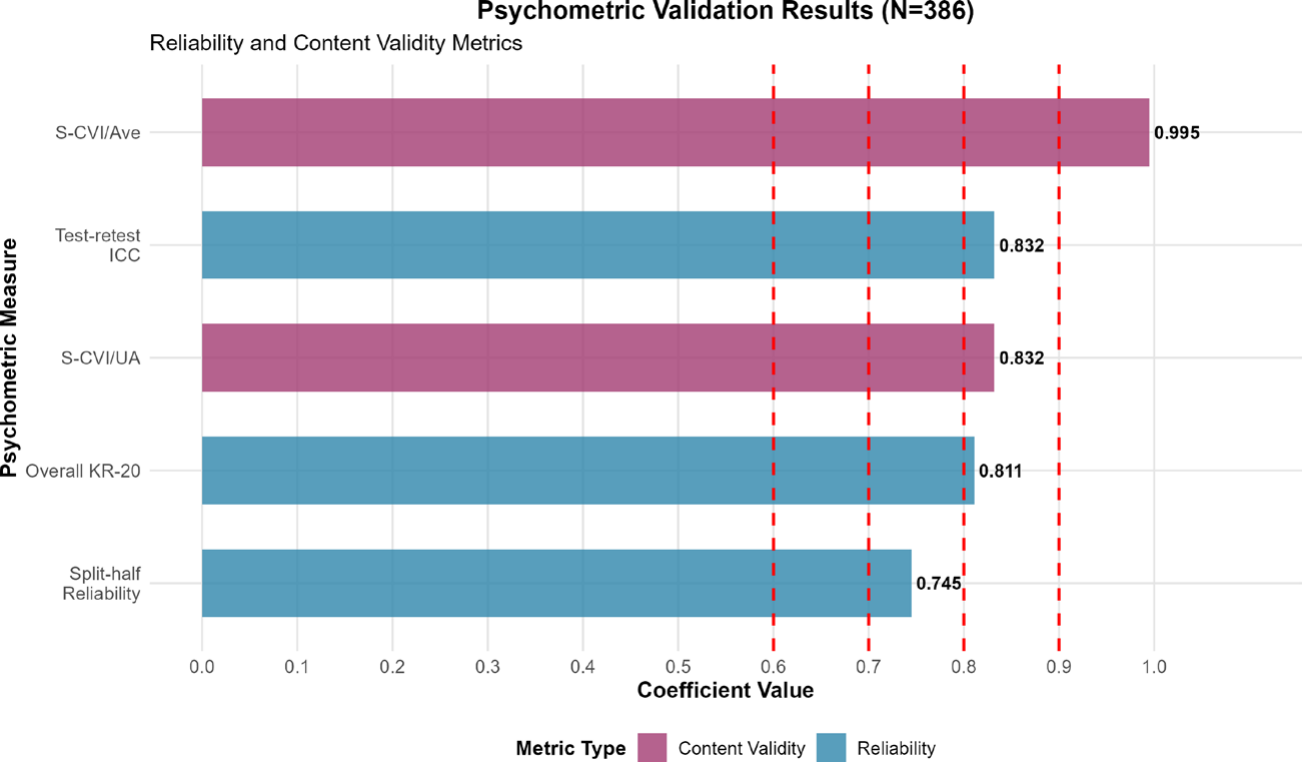
Psychometric validation results (N = 386). Reliability and content validity metrics demonstrating robust psychometric properties. All coefficients exceeded established thresholds (red dashed lines), with overall KR-20 = 0.811, split-half reliability = 0.745, test–retest ICC = 0.832, S-CVI/Ave = 0.995, and S-CVI/UA = 0.832. I-CVI = Item-Level Content Validity Index, KR-20 = Kuder–Richardson Formula 20, S-CVI/Ave = Scale-Level Content Validity Index Average, S-CVI/UA = Scale-Level Content Validity Index Universal Agreement.

Test–retest reliability was assessed in a subsample of 45 participants over a 2-week interval, yielding an intraclass correlation coefficient of 0.832 (95% CI: 0.751–0.891), demonstrating excellent temporal consistency (Table 3, Fig. 3).

**Table 3:** Validation sample characteristics and performance metrics.

Characteristic	Value
Sample demographics (n .= 386)	
Age (yr), mean ± SD	14.6 ± 2.1
Age range	11–18 years
Gender, n (%)	
Female	198 (51.3)
Male	188 (48.7)
Clinical characteristics	
Primary diagnosis, n (%)	
Major depressive disorder	312 (80.8)
Persistent depressive disorder	48 (12.4)
Other specified depressive disorder	26 (6.7)
Assessment period	June-September 2024
Reliability metrics	
Overall KR-20	0.811
Split-half reliability	0.745
Test–retest ICC (n = 45)	0.832 (95% CI: 0.751–0.891)
Test–retest interval	2 weeks
Content validity metrics	
Number of expert evaluators	7
I-CVI range	0.881–1.000
Items with perfect I-CVI (1.000), n (%)	34 (89.5)
Items with I-CVI ≥ 0.90, n (%)	38 (100.0)
S-CVI/Ave	0.995
S-CVI/UA	0.832
Inter-rater reliability	
Dual-coded assessments, n (%)	39 (10.1)
Cohen Kappa range	0.80–0.95
Mean Cohen Kappa	0.87 ± 0.04

I-CVI = Item-Level Content Validity Index, KR-20 = Kuder–Richardson Formula 20, S-CVI/Ave = Scale-Level Content Validity Index Average, S-CVI/UA = Scale-Level Content Validity Index Universal Agreement.

### 3.6. Content validity assessment

Content validity evaluation was conducted by 7 randomly selected experts from the consultation panel, ensuring independent assessment of indicator relevance and appropriateness. These experts were different from those involved in the Delphi rounds, providing unbiased content validity evaluation. The I-CVI demonstrated exceptional performance, with values ranging from 0.881 to 1.000 across all indicators.

Detailed analysis revealed that 34 of 38 secondary indicators (89.5%) achieved perfect I-CVI scores of 1.000, while the remaining 4 indicators scored between 0.881 and 0.943, all substantially exceeding the required threshold of ≥ 0.78 (Table 2). The S-CVI/Ave reached 0.995, far surpassing the required threshold of ≥ 0.90 and indicating exceptional overall content validity. The S-CVI/UA achieved 0.832, exceeding the required threshold of ≥ 0.80 and confirming strong cross-expert consensus regarding indicator appropriateness (Table 3, Fig. 3).

### 3.7. Final risk warning indicator system architecture

**Figure 4.**
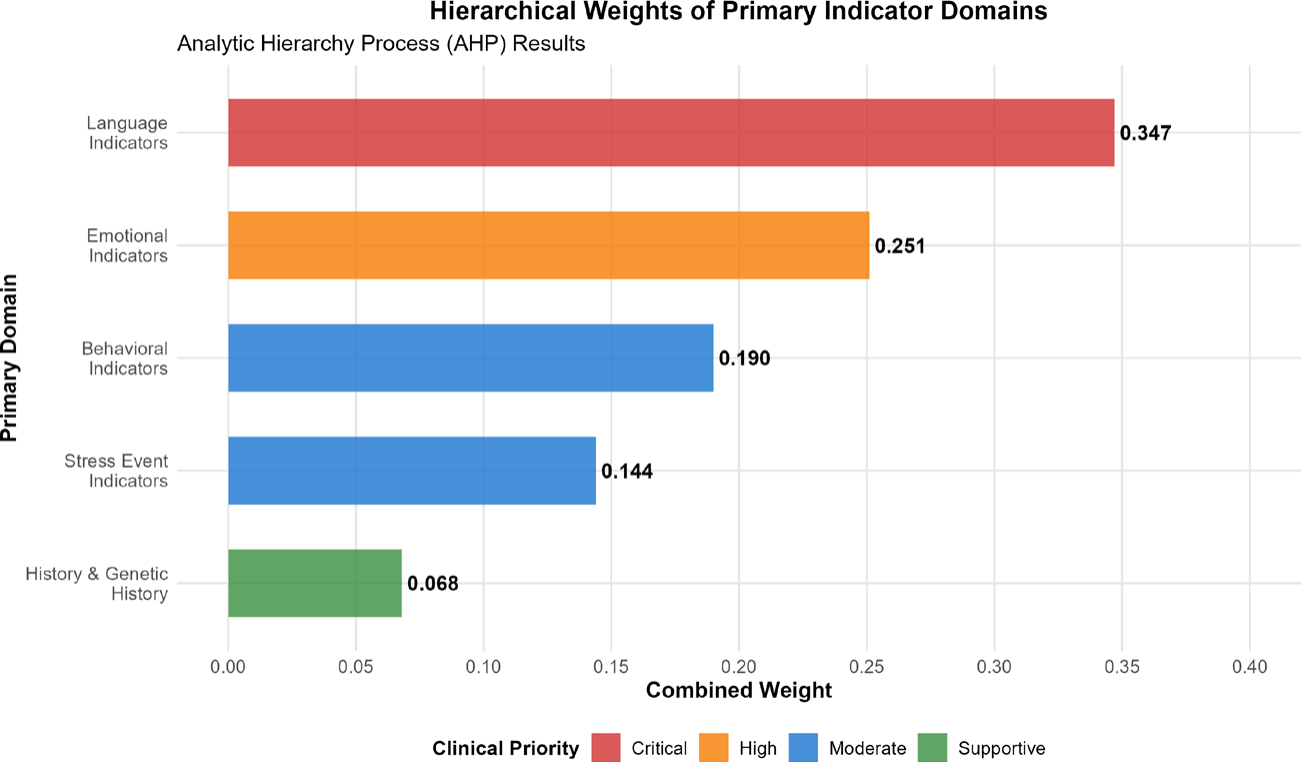
Hierarchical weights of primary indicator domains. Combined weights derived through analytic hierarchy process (AHP) methodology, showing the relative clinical importance of each primary domain. Language indicators received the highest weight (0.347), reflecting their critical role in early depression detection, followed by emotional (0.251) and behavioral (0.190) indicators.

The comprehensive development and validation process culminated in a sophisticated 5-domain indicator system comprising 38 secondary indicators (Table 2, Fig. 4). The final system demonstrated optimal balance between comprehensiveness and clinical utility, with each domain contributing distinct but complementary risk assessment capabilities.

Language indicators emerged as the most heavily weighted domain (weight = 0.347), encompassing 9 secondary indicators that capture verbal expressions of distress, suicidal ideation, and somatic complaints (Table 2). This domain’s prominence reflects the critical importance of verbal communication in identifying depression risk among children and adolescents. Among the secondary indicators, “uncontrollably revealing suicidal ideation” achieved the highest combined weight of 0.084, serving as the most critical warning signal within this domain.

**Figure 5.**
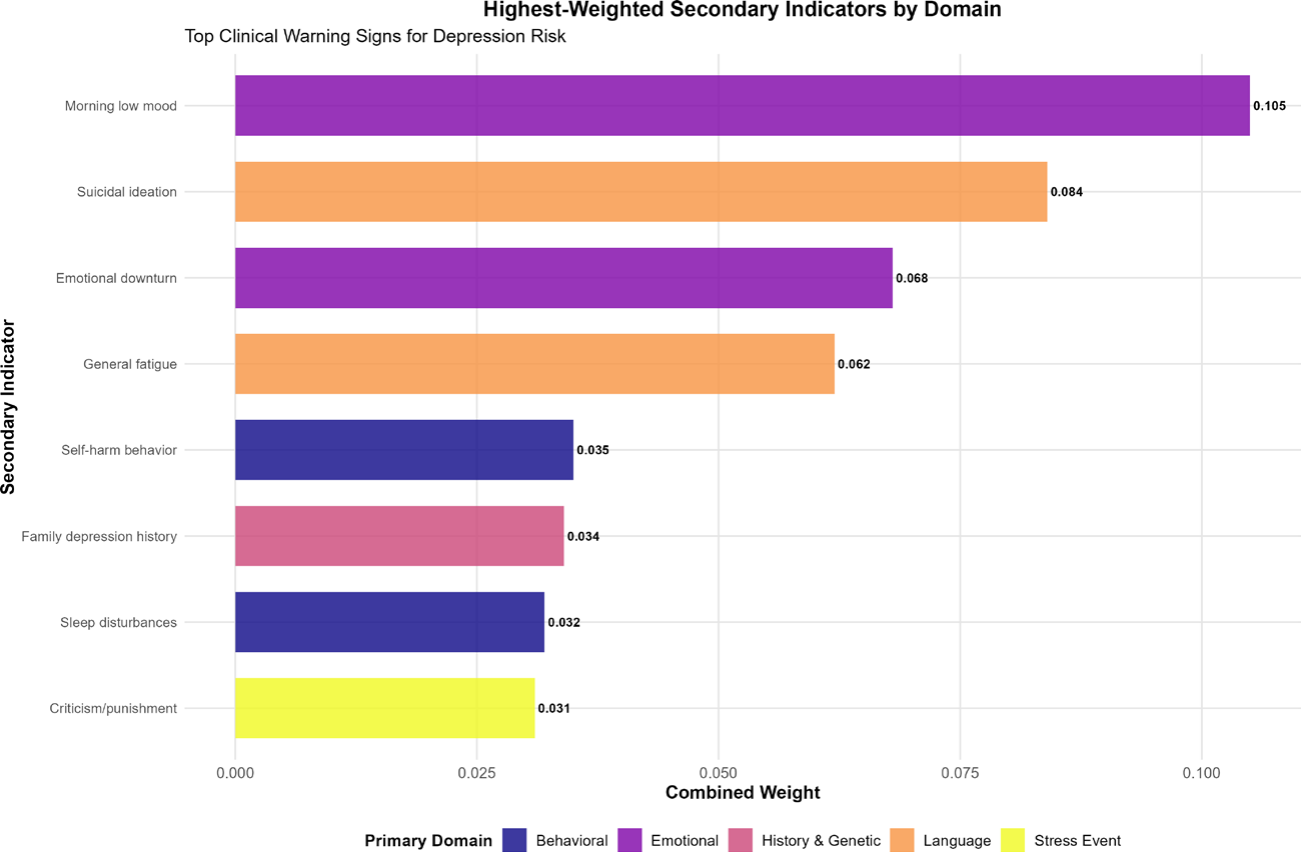
Highest-weighted secondary indicators by primary domain. Top-ranking secondary indicators representing the most critical clinical warning signs for depression risk assessment. “Morning low mood” achieved the highest individual weight (0.105), followed by “suicidal ideation” (0.084), and “emotional downturn” (0.068).

Emotional indicators constituted the second-ranked domain (weight = 0.251) with 8 secondary indicators addressing mood patterns, emotional regulation difficulties, and affective symptoms (Table 2, Fig. 4). The inclusion of diurnal mood variation and persistent emotional downturn as primary indicators reflects established clinical patterns in pediatric depression. The indicator “morning low, evening high mood pattern” achieved the highest combined weight of 0.105 within this domain, representing a pathognomonic feature of depression (Fig. 5).

Behavioral indicators represented the third-weighted domain (weight = 0.190) with 10 secondary indicators encompassing self-harm behaviors, sleep disturbances, social withdrawal, and behavioral changes (Table 2, Fig. 4). This domain’s comprehensive coverage addresses both internalizing and externalizing behavioral manifestations of depression. “Self-harming behavior” emerged as the highest-weighted indicator within this domain with a combined weight of 0.035 (Fig. 5).

Stress event indicators formed the fourth domain (weight = 0.144) with 9 secondary indicators capturing recent environmental stressors, interpersonal conflicts, and traumatic experiences (Table 2, Fig. 4). The 3-month timeframe for stress events reflects evidence-based understanding of acute stressor impact on adolescent mental health. “Being criticized or punished” achieved the highest combined weight of 0.031 within this domain (Fig. 5).

History and genetic history indicators comprised the fifth domain (weight = 0.068) with 2 secondary indicators addressing family psychiatric history and personal trauma exposure (Table 2). Despite lower weighting, this domain provides crucial contextual information for comprehensive risk assessment. “Direct relatives with depression” achieved the highest combined weight of 0.034 within this domain.

The hierarchical weighting system was derived through AHP methodology, with consistency ratios for all pairwise comparison matrices below 0.10, confirming mathematical validity of the weight assignments.

## 4. Discussion

The development of a scientifically robust and clinically applicable risk warning indicator system for depression in children and adolescents represents a critical advancement in pediatric mental health care, addressing a significant gap in early detection capabilities for one of the most prevalent and devastating mental health conditions affecting young populations. Recent trends demonstrate an alarming increase in mental, behavioral, and developmental disorders among children aged 3 to 17 years, with prevalence rising from 25.3% to 27.7% between 2016 and 2021, with particularly notable increases in anxiety and depression.^[1]^ This study successfully established a comprehensive 5-domain framework comprising 38 secondary indicators through rigorous methodological approaches that combine systematic evidence synthesis, expert consensus methodology, and empirical validation. The resulting indicator system demonstrates exceptional psychometric properties while providing a practical tool for healthcare professionals, educators, and families to identify at-risk youth before the onset of severe symptomatology.

The methodological rigor employed throughout this investigation ensures both scientific validity and practical applicability, distinguishing this work from previous efforts in the field. The 2-round Delphi consultation process engaged 20 distinguished experts from 8 tertiary psychiatric hospitals across 6 provinces, achieving unanimous participation with Ca substantially exceeding established thresholds. This geographic and professional diversity strengthens the generalizability of findings while ensuring that the indicator system reflects contemporary clinical understanding across different healthcare contexts. The progressive improvement in expert authority metrics from the first to second consultation rounds (Ca = 0.808 to 0.868), coupled with statistically significant coordination coefficients (W = 0.131 and 0.142, both *P* < .001), confirms the reliability and credibility of the consensus-building process. Such methodological excellence is particularly crucial given the complexity of adolescent depression presentation and the need for indicators that can be consistently applied across diverse clinical and educational settings, especially considering that current evidence suggests screening instruments for depression demonstrate sensitivity ranging from 0.59 to 0.94 and specificity from 0.38 to 0.96.^[4,19]^

The hierarchical weighting structure derived through AHP methodology reveals important insights into the relative clinical significance of different manifestation domains, providing evidence-based guidance for prioritizing assessment focus. Language indicators emerged as the most heavily weighted domain (weight = 0.347), reflecting the fundamental importance of verbal communication in identifying depression risk among children and adolescents. This finding aligns with established clinical understanding that verbal expressions of distress, suicidal ideation, and somatic complaints often represent the earliest and most accessible warning signs for healthcare professionals, educators, and family members. The prominence of “uncontrollably revealing suicidal ideation” as the highest-weighted individual indicator (combined weight = 0.084) underscores the critical nature of verbal suicide risk communication and reinforces the necessity for immediate intervention protocols when such expressions are identified. This emphasis on language indicators is particularly relevant in the Chinese cultural context, where direct emotional expression may be less common, making verbal indicators of distress especially significant for early detection efforts. Research has consistently demonstrated that higher cognitive functioning and capacity for introspection in adolescents with depression increases their ability to articulate internal distress, making language-based indicators particularly valuable for early identification.^[6,10]^

The second-ranked emotional indicators domain (weight = 0.251), encompassing mood patterns and affective symptoms, demonstrates the central role of emotional dysregulation in adolescent depression. The identification of “morning low, evening high mood pattern” as the highest-weighted individual indicator across all domains (combined weight = 0.105) provides valuable clinical guidance for recognizing pathognomonic features of depression. This diurnal mood variation represents a specific and observable pattern that can be readily identified by parents, teachers, and healthcare providers, facilitating early detection efforts. The substantial weight assigned to “persistent emotional downturn” (combined weight = 0.068) further emphasizes the importance of sustained mood changes as distinguished from transient emotional fluctuations common in typical adolescent development. These findings support the integration of systematic mood monitoring into routine assessment protocols and highlight the importance of educating caregivers about recognizable emotional patterns that warrant professional attention. Contemporary research emphasizes that negative emotions serve as mediators between stress factors and suicidal ideation, making emotional indicators particularly crucial for comprehensive risk assessment.^[20]^

Behavioral indicators, while ranking third in overall domain weight (weight = 0.190), encompass critical manifestations that often prompt initial clinical attention and provide observable evidence of internal distress. The prominence of self-harming behavior within this domain (combined weight = 0.035) reflects the serious nature of these presentations and their strong association with suicide risk. Sleep disturbances and eating disorders, ranking second within behavioral indicators (combined weight = 0.032), highlight the bidirectional relationship between physical health behaviors and mental health in adolescent populations. These findings support the integration of comprehensive behavioral assessment into routine depression screening protocols and emphasize the importance of addressing sleep hygiene and eating patterns as both preventive and therapeutic interventions.^[10]^ The inclusion of perceptual disturbances and inconsistencies between behavior and inner feelings as secondary indicators acknowledges the complex phenomenology of adolescent depression and provides guidance for identifying subtle behavioral changes that may precede more obvious symptomatology. Emerging research on gut microbiota-based interventions suggests that dietary factors and gastrointestinal health may play significant roles in depression and anxiety among children and adolescents, further supporting the inclusion of eating-related behavioral indicators.^[3,21]^

The inclusion of stress event indicators (weight = 0.144) acknowledges the significant role of environmental factors in adolescent depression onset and progression, reflecting contemporary understanding of the multifactorial nature of mental health conditions. The 3-month timeframe for stress event assessment reflects evidence-based understanding of acute stressor impact while maintaining practical applicability for clinical assessment. The prominence of criticism and punishment as the highest-weighted stress indicator (combined weight = 0.031) provides important guidance for educational and parenting approaches, suggesting that excessive criticism may contribute to depression risk and should be balanced with supportive interventions. The identification of school bullying as a significant stress factor (combined weight = 0.024) aligns with international research demonstrating the substantial impact of peer victimization on adolescent mental health outcomes and reinforces the importance of comprehensive anti-bullying initiatives in educational settings. Recent epidemiological data indicate that stressful life events, particularly those involving interpersonal relationships and academic pressures, represent significant risk factors for depression onset in adolescent populations.^[3,4,22]^

History and genetic history indicators, while receiving the lowest domain weight (weight = 0.068), provide essential contextual information for comprehensive risk assessment and long-term monitoring strategies. The identification of family depression history as the most significant factor within this domain (combined weight = 0.034) aligns with established genetic and environmental transmission patterns of depression. This domain’s inclusion ensures that the indicator system captures both immediate presentation features and underlying vulnerability factors that may influence treatment planning and prevention strategies. The recognition of trauma and abuse history as significant risk factors (combined weight = 0.021) acknowledges the well-established relationship between adverse childhood experiences and mental health outcomes, providing guidance for trauma-informed assessment approaches. Research consistently demonstrates that individuals with family histories of mental disorders exhibit stronger susceptibility, longer disease courses, and more prominent anxiety symptoms, necessitating enhanced monitoring and intervention strategies.^[3,14,23]^

The psychometric validation results demonstrate exceptional reliability and content validity characteristics that exceed established standards for clinical assessment instruments, providing confidence in the instrument’s scientific rigor and clinical utility. The overall KR-20 coefficient of 0.811 indicates strong internal consistency across all indicators, while the split-half reliability coefficient of 0.745 confirms temporal stability. The test–retest reliability coefficient of 0.832 (95% CI: 0.751–0.891) demonstrates excellent consistency over time, supporting the instrument’s utility for longitudinal monitoring. The content validity indices, with 89.5% of indicators achieving perfect scores of 1.000, reflect the high relevance and appropriateness of individual items as assessed by independent expert evaluators. The scale-level content validity index average of 0.995 and universal agreement index of 0.832 confirm strong cross-expert consensus regarding indicator appropriateness and clinical relevance. These robust psychometric properties provide confidence in the instrument’s ability to accurately assess depression risk while minimizing false positive and false negative classifications, which is particularly important given that current screening approaches demonstrate variable accuracy across different populations.^[4,9]^

The clinical implications of this indicator system extend beyond individual risk assessment to encompass broader public health applications and systematic approaches to mental health promotion. The translation of complex psychological symptoms into observable, assessable indicators enable non-specialist identification of at-risk youth, potentially facilitating earlier intervention and improved outcomes. The system’s design allows for implementation across multiple settings, including schools, primary care practices, and community mental health programs, thereby expanding the reach of depression screening efforts and supporting integrated care approaches. The hierarchical weighting structure provides evidence-based guidance for resource allocation and intervention prioritization, enabling healthcare systems to focus attention on the most critical warning signs while maintaining comprehensive assessment capabilities. This approach is particularly valuable considering that approximately 60% of children with mental, behavioral, and developmental disorders receive appropriate services annually, indicating substantial gaps in current identification and treatment systems.^[10]^

The international context of adolescent depression research provides important perspective on the significance of these findings. Recent bibliometric analyses demonstrate that research on depression among children and adolescents has gained substantial momentum globally, with particular emphasis on addressing comorbidities, adverse childhood experiences, and health inequalities.^[10]^ The IDEA project in Brazil and similar initiatives in the United Kingdom have demonstrated the feasibility and importance of developing culturally appropriate risk assessment tools for adolescent populations, with prediction models showing promising performance across different continental contexts. However, the cultural specificity of depression presentation and the unique characteristics of the Chinese healthcare and educational systems necessitate locally developed and validated assessment tools, making this study’s contribution particularly valuable for Asian populations. Additionally, emerging research on lifestyle factors, including ultra-processed food consumption and nutritional interventions such as omega-3 fatty acid supplementation, suggests that comprehensive risk assessment should incorporate environmental and dietary factors that may influence depression risk.^[4,6,24,25]^

Several limitations warrant consideration in interpreting these findings and planning future research directions. The indicator development process, while methodologically rigorous, necessarily involves subjective expert judgment that may reflect current clinical paradigms and cultural contexts specific to the Chinese healthcare system. The validation sample, drawn from a single healthcare institution, may limit generalizability to other populations or healthcare contexts, particularly those with different demographic characteristics or healthcare delivery models. Additionally, the cross-sectional validation design precludes assessment of the indicator system’s predictive validity or sensitivity to change over time, limiting understanding of the system’s utility for monitoring treatment response or predicting clinical outcomes. Current evidence regarding depression treatment effectiveness demonstrates that psychotherapy and pharmacotherapy show benefits for symptom reduction and functional improvement, but limited data exist regarding the long-term predictive value of screening instruments.^[4]^

The temporal stability of the indicator system requires ongoing evaluation as understanding of adolescent depression continues to evolve and new research emerges regarding risk factors and protective mechanisms. Emerging research on neurobiological markers, digital behavioral indicators, and social media-based risk factors may necessitate future refinements to maintain contemporary relevance and incorporate technological advances in mental health assessment. Additionally, the system’s performance across different demographic subgroups, including various ethnic, socioeconomic, and cultural populations within China, requires systematic evaluation to ensure equitable application and avoid potential bias in risk assessment procedures. Recent research on specialized populations, such as children and adolescents with autism spectrum disorders, highlights the need for adapted assessment approaches that consider unique risk factors and symptom presentations.^[6]^

Implementation considerations include the need for comprehensive training programs for potential users, development of standardized administration protocols, and establishment of referral pathways for positive screenings. The integration of this indicator system into existing clinical workflows and educational settings will require careful planning and stakeholder engagement to maximize adoption and effectiveness while minimizing implementation burden. The development of digital platforms and mobile applications to support indicator assessment and scoring could enhance accessibility and standardization of the assessment process. Given that unmet healthcare needs among children with mental health conditions have increased annually by approximately 5%, systematic implementation of validated screening tools represents a critical public health priority.^[15]^

Future research priorities should focus on prospective validation studies across diverse populations and healthcare settings to establish predictive accuracy and clinical utility. Longitudinal studies examining the relationship between indicator scores and clinical outcomes would provide valuable evidence regarding the system’s prognostic value and inform refinements to improve predictive performance. Additionally, intervention studies examining the impact of early identification using this indicator system on clinical outcomes and healthcare utilization would provide important evidence regarding the system’s public health value and cost-effectiveness. Research exploring the integration of emerging technologies, including digital biomarkers and artificial intelligence-based assessment tools, could enhance the system’s precision and accessibility.^[6]^

## 5. Conclusion

This investigation establishes a scientifically validated foundation for early depression detection in children and adolescents while providing a framework for systematic risk assessment across multiple domains. The indicator system’s robust psychometric properties, combined with its practical applicability and cultural appropriateness, position it as a valuable tool for advancing pediatric mental health care in China and potentially other Asian populations. The hierarchical weighting structure provides evidence-based guidance for prioritizing assessment focus while maintaining comprehensive coverage of depression manifestations. As healthcare systems increasingly emphasize preventive approaches to mental health care, this indicator system offers a systematic methodology for identifying at-risk youth and facilitating timely intervention. The successful development and validation of this comprehensive risk warning system represents a significant contribution to pediatric mental health assessment capabilities and establishes a foundation for future research and clinical application efforts in the prevention and early intervention of adolescent depression.

## Author contributions

**Conceptualization:** Yan Han, Jingrong Xi, Chunhong Wei.

**Data curation:** Yan Han, Chunhong Wei.

**Formal analysis:** Yan Han, Chunhong Wei.

**Funding acquisition:** Chunhong Wei.

**Investigation:** Chunhong Wei.

**Methodology:** Jingrong Xi, Chunhong Wei.

**Project administration:** Chunhong Wei.

**Resources:** Yan Han, Jingrong Xi, Chunhong Wei.

**Software:** Yan Han, Chunhong Wei.

**Supervision:** Huizhu Jiang.

**Validation:** Huizhu Jiang, Chunhong Wei.

**Visualization:** Chunhong Wei.

**Writing – original draft:** Yan Han, Chunhong Wei.

**Writing – review & editing:** Jingrong Xi, Huizhu Jiang, Chunhong Wei.
